# Pathogenesis, Early Diagnosis, and Therapeutic Management of Alcoholic Liver Disease

**DOI:** 10.3390/ijms20112712

**Published:** 2019-06-02

**Authors:** Ling-Zu Kong, Nisansala Chandimali, Ying-Hao Han, Dong-Ho Lee, Ji-Su Kim, Sun-Uk Kim, Tae-Don Kim, Dong Kee Jeong, Hu-Nan Sun, Dong Sun Lee, Taeho Kwon

**Affiliations:** 1 Laboratory of Animal Genetic Engineering and Stem Cell Biology, Advanced Convergence Technology and Science, Jeju National University, Jeju 63243, Korea; konglingzu93@gmail.com (L.-Z.K.); nimminisha28@gmail.com (N.C.); newdkjeong@gmail.com (D.K.J.); 2 Immunotherapy Convergence Research Center, Korea Research Institute of Bioscience and Biotechnology (KRIBB), Daejeon 34141, Korea; tdkim@kribb.re.kr; 3 Department of Disease Model Animal Research Center, College of Life Science and Technology, Heilongjiang Bayi Agricultural University, Daqing 163319, China; huyhbynd@163.com; 4 Primate Resources Center, Korea Research Institute of Bioscience and Biotechnology (KRIBB), Jeongeup-si, Jeonbuk 56216, Korea; luckyberry@kribb.re.kr (D.-H.L.); kimjs@kribb.re.kr (J.-S.K.); 5 Futuristic Animal Resource & Research Center, Korea Research Institute of Bioscience and Biotechnology (KRIBB), Cheongju-si, Chungcheongbuk-do 28116, Korea; sunuk@kribb.re.kr; 6 Subtropical/Tropical Organism Gene Bank, Jeju National University, Jeju 63243, Korea; 7 Department of Biotechnology, College of Applied Life Science, Jeju National University, Jeju 63243, Korea

**Keywords:** ALD, pathogenesis, diagnose, phytochemical, MSCs

## Abstract

Alcoholic liver disease (ALD) refers to the damages to the liver and its functions due to alcohol overconsumption. It consists of fatty liver/steatosis, alcoholic hepatitis, steatohepatitis, chronic hepatitis with liver fibrosis or cirrhosis, and hepatocellular carcinoma. However, the mechanisms behind the pathogenesis of alcoholic liver disease are extremely complicated due to the involvement of immune cells, adipose tissues, and genetic diversity. Clinically, the diagnosis of ALD is not yet well developed. Therefore, the number of patients in advanced stages has increased due to the failure of proper early detection and treatment. At present, abstinence and nutritional therapy remain the conventional therapeutic interventions for ALD. Moreover, the therapies which target the TNF receptor superfamily, hormones, antioxidant signals, and MicroRNAs are used as treatments for ALD. In particular, mesenchymal stem cells (MSCs) are gaining attention as a potential therapeutic target of ALD. Therefore, in this review, we have summarized the current understandings of the pathogenesis and diagnosis of ALD. Moreover, we also discuss the various existing treatment strategies while focusing on promising therapeutic approaches for ALD.

## 1. Introduction

Alcoholic liver disease (ALD) is the second most common cause of total human death every year [1]. According to previous studies, ALD includes alcoholic fatty liver, alcoholic hepatitis, steatohepatitis, liver fibrosis, cirrhosis, and liver cancer [2]. Long-term excessive drinking can cause oxidative stress in the liver through the accumulation of reactive oxygen species (ROS) produced from alcohol metabolism [3]. It causes liver inflammation through the toxicity of lipopolysaccharides (LPS) and acetaldehyde. Moreover, the burden to the liver can be increased by other symptoms, such as chronic viral hepatitis and nonalcoholic fatty liver [4,5]. Fat accumulation in the liver, an early stage of ALD, is the only stage of ALD which can be completely reversible through abstinence without any drug intervention. Therefore, the diagnosis of early stage ALD and treatment with correct strategies is pivotal to treat ALD successfully if irreversible liver damage has not yet occurred [6].

Even though ALD has a profound impact on public health, the research progress of this field stands at a relatively low level. Furthermore, the pathogenesis of the disease remains unclear. To date, alcohol abstinence and nutrient supplements are considered the conventional treatment strategies for ALD in the clinic [7]. Therefore, the current researchers have focused on more therapeutic strategies, such as improving patient survival and prognosis, prevention of ALD and early diagnosis and targeted therapies [7]. As a result, the accumulating evidence has shown that the effects of non-toxic compounds extracted from natural food and herbal plants prevent ALD [8]. Especially, it has also shown the potential application of mesenchymal stem cells (MSCs)-based therapies for ALD treatment [9,10].

In this review paper, we summarized the recent studies on pathogenesis, underlying mechanisms, and diagnosis of ALD. In addition, we also summarized the possible therapeutic strategies to treat or prevent ALD.

## 2. Pathogenesis of ALD

### 2.1. Alcohol Metabolism

Alcohol, a polar molecular substance, is soluble in both water and lipid. After being consumed, it is absorbed into the blood circulation through the gastrointestinal tract. Generally, more than 95% of absorbed alcohol is metabolized by the liver, and a small amount is directly excreted through the lungs, urine, and sweat [11].

As shown in previous studies, there are three main metabolic systems which participate in the oxidative metabolism of alcohol into acetaldehyde. First is the hepatocyte cytoplasmic alcohol dehydrogenase (ADH) system, which oxidizes ethanol into acetaldehyde using nicotinamide adenine dinucleotide (NAD^+^) as a co-factor. ADH1, amongst the other isoenzymes of ADH, plays a main role in ethanol metabolism in the liver. Acetaldehyde, which is produced by ADH, is further oxidized to acetate by acetaldehyde dehydrogenase (ALDH) [12,13].

The second main metabolic system of alcohol is cytochrome P450 2E1 (CYP2E1) enzymes that convert chemical molecules into polar metabolites prior to excretion. CYP2E1 is considered to be the main component of this system. Under normal physiological conditions, CYP2E1 catalyzes the oxidization of a small amount of ethanol, such as 10%, into acetaldehyde. However, it is increased in chronic alcohol abuse conditions due to the induction of the expression of CYP2E1. The third metabolic system is the catalase endoplasmic reticulum (CAT) system, which is considered as the other main metabolic system that relies on NADPH for oxidative metabolism. This heme-containing catalase enzyme usually catalyzes the H2O2 removal but also is capable of catalyzing the oxidation of alcohol to acetaldehyde [14,15].

When the concentration of ethanol in blood and tissue fluid is low, it is mainly metabolized by the ADH pathway. However, when the concentration of ethanol is higher than 10 mol/L, the other two enzyme systems also begin to participate in ethanol metabolism [15].

### 2.2. The Spectrum of ALD

ALD comprises a broad spectrum of liver disorders ranging from simple alcoholic fatty liver/steatosis to severe lesion of liver injuries including steatohepatitis, fibrosis/cirrhosis, and hepatocellular carcinoma. These stages are classified based on the histology of the liver of patients [16]. Frequently, the pathologic processes are overlapped instead of being distinct disorder entities [17,18].

#### 2.2.1. Alcoholic Fatty Liver (AFL)/Steatosis

Fatty liver, the earliest liver response to alcohol abuse, is generally able to revert completely by abstinence or maintained by the moderation in alcohol consumption. In this stage, fat vacuoles can be observed in liver tissue sections under the microscope [5]. According to previous studies, the over-consumption of alcohol increases the ratio of reduced nicotinamide adenine dinucleotide to oxidized nicotinamide adenine dinucleotide in hepatocytes. It results in AFL by disrupting the mitochondrial β-oxidation of fatty acids [5,19].

**Figure 1:**
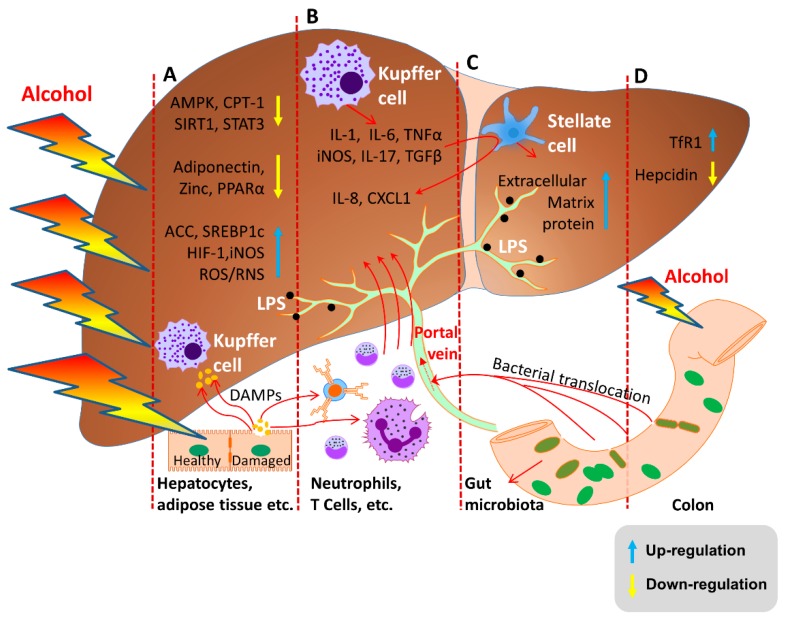
Molecular mechanisms of ALD. (**A**) Alcohol and its metabolites cause AFL by increasing ROS/RNS levels and the expression of ACC and SREBP1c. Additionally, via the reduced expression of AMPK-SIRT1, adiponectin, and zinc which activate PPARα. (**B**,**C**) Excessive alcohol consumption-enhanced permeability of the colon allows LPS to enter into the liver through the portal vein. The activated Kupffer cells release cytokines such as IL-1, IL-17, TGF-β, iNOS, and TNF-α which activates stellate cells and release IL-8 and CXCL1 in AH and ASH. Activated stellate cells also release the extracellular matrix which results in liver fibrosis. (**D**) Regulation of hepcidin, one of the main pathogenic factors in ALD.

Moreover, recent studies have shown several underlying mechanisms which develop AFL through the direct or indirect regulation of lipid-metabolism associated factors. According to those studies, alcohol abuse increases sterol regulatory element-binding proteins c (SREBP-1c) expression and diminishes peroxisome proliferator-activated receptor alpha (PPAR-α) expression which leads the development of AFL via the induction of fatty acid synthesis and the inhibition of fatty acid β-oxidation. Furthermore, the consumption of alcohol subsequently induces Acetyl-CoA Carboxylase (ACC) activity and suppresses carnitine palmitoyltransferase I (CPT-1) activity via inhibiting AMP-activated protein kinase (AMPK) which develop AFL by increased fatty acid synthesis and reduced fatty acid β-oxidation. Furthermore, alcohol modulates many factors, such as the hypoxia-inducible Factor 1 (HIF-1) and inducible nitric oxide synthase (iNOS), which contribute to AFL development [5,15,20,21,22] (Figure 1).

After AFL develops, and if the person does not get treatment, fibrosis and cirrhosis may develop, followed by eventual liver failure [5].

#### 2.2.2. Alcoholic Hepatitis (AH)

AH, a clinical syndrome of liver function decompensation, develops in patients with an ALD related background [23]. AH is not directly related to an alcohol dose. Therefore, only 10 to 35% of heavy drinkers develop AH. AH is associated with poor liver function and ductular formation. Furthermore, in previous studies, induced levels of LPS and impaired hepatocyte proliferation have been shown to be characteristics of AH [24,25] (Figure 1).

#### 2.2.3. Alcoholic Steatohepatitis (ASH)

ASH is characterized by the hepatic injuries associated with steatosis. Most commonly, the hepatocyte ballooning can be identified as the frequent cellular injury of the ASH [26,27]. Studies have shown that alcohol and obesity can promote the development of steatohepatitis. The inflammatory environment produced in alcoholic liver subsequently leads to polymorph nuclear leukocyte infiltration, ROS formation, and hepatocyte damage. Finally, the damaged proteins are degraded by proteasome pathways which increase endoplasmic reticulum (ER) stress, alter autophagy, and promote hepatocyte injury. It also induces the development of hepatic inclusions that aggregate cytokeratin, which is known as the Mallory–Denk body [4,28].

When sterile inflammation (SI) occurs, damage-associated molecular patterns (DAMPs) can be released from damaged tissues. It can activate receptors on immune cells to promote the development of liver fibrosis and cancer [29] (Figure 1).

#### 2.2.4. Alcoholic Liver Fibrosis/Cirrhosis

Heavy alcohol consumption is associated with the fibrosis progression in other types of liver diseases. The acetaldehyde produced by the oxidation of ethanol has a high toxicity and high activity which can destroy the microtubule structure of hepatocyte, causing microtubule dysfunction and further affecting the transportation of nutrients. Moreover, the formation of acetaldehyde-protein adducts further leads the protease inactivation of hepatocytes, abnormalities in DNA repair, damages in hepatocyte mitochondrial structure, oxygen utilization disorders, stimulation of collagen synthesis, and the accumulation of extracellular matrix proteins to form liver fibrosis and cirrhosis (Figure 1). Activation of the hepatic stellate cell (HSC) is the key step in the pathogenesis of alcoholic liver fibrosis (Figure 1). Dependent on these understandings; bone marrow cells (BMC), hematopoietic stem cells (HSC), and mesenchymal stem cells (MSC) have been used recently to develop cirrhosis treatment [30,31].

### 2.3. Molecular Mechanisms of ALD

During the last decade, significant effort has been made to understand the underlying molecular mechanisms of ALD which contribute to its pathogenesis [32]. According to previous studies, those mechanisms include direct hepatotoxicity, alcohol and its metabolites induced ROS production, the activation of innate immunity, and the production of pro-inflammatory cytokines [32,33,34].

#### 2.3.1. Alcohol and Its Metabolites Damage the Liver

As a primary metabolite of alcohol, acetaldehyde has obvious toxic effects on liver by damaging the mitochondria and microtubules of hepatocytes. Generally, CYP2E1 is up-regulated in chronic alcohol abuse conditions and leads to converting more alcohol to acetaldehyde [35]. Acetaldehyde causes the dysfunction of mitochondrial fatty acid β-oxidation and hepatocyte secretion. Furthermore, it directly activates the transcription of SREBP-1c to promote the synthesis of fatty acids which results in fatty acid accumulation in hepatocytes [5] (Figure 1).

In chronic alcohol consumption, CYP2E1 enzyme is absolutely responsible for the excessive production of ROS, considered the second messenger in the cell. These produced ROS, such as hydrogen peroxide and superoxide ions, are associated with the pro-inflammatory profile of alcohol-mediated liver damages via recruiting immune cells and inducing pro-inflammatory cytokines circulation [35,36]. Moreover, in fatty liver disease, ROS-induced oxidative stress can inhibit the expression of energy metabolism signaling pathway-related proteins such as AMPK, Sirtuin 1 (SIRT1), and the transcription factor signal transducer and activator of transcription 3 (STAT3) [37] (Figure 1)**.** It also down-regulates the levels of adiponectin and zinc that activates PPARα [5] (Figure 1), causing the peroxidation of hepatic membrane phospholipids, producing a large number of lipid free radicals, and promoting the expression of early growth response protein 1 (Egr-1), tumor necrosis factor alpha (TNF-α), adiponectin and acetyl-CoA carboxylase (ACC), which cause the accumulation of fat in liver [5].

#### 2.3.2. Oxidative Stress and Lipid Peroxidation

In chronic alcohol abuse, alcohol increases the expression of CYP2E1 in liver, causing dysregulations in lipid peroxidation through ROS free radicals. Therefore, it leads to the dynamic imbalance of antioxidant systems which target the important antioxidant regulatory gene nuclear factor erythroid 2-related factor 2 (Nrf-2) in ALD [38]. It also damages other antioxidant genes which have important roles in ROS scavenging, such as superoxide dismutase (SOD), glutathione (GSH), catalase, peroxidase-1, metallothionein, and heme oxygenase (HO-1) [39,40]. Studies have shown that peroxiredoxin-1 and CYP2E1 located in the cytoplasmic side of the ER membrane can directly remove the ROS produced by the metabolism of alcohol. However, the expression of these antioxidant proteins is regulated by Nrf-2 or Thioredoxin (Trx) [41]. Therefore, the removal of ROS is dysregulated by damaged Nrf-2.

#### 2.3.3. Endotoxin Enteric Leakage

Research has shown that chronic drinking can cause the imbalance of intestinal flora and intestinal toxin accumulation. Alcohol exposure causes the excessive proliferation of gram negative bacteria in the intestine. This growth results in the accumulation of endotoxin. Other than this, the metabolism of alcohol by gram negative bacteria and intestinal epithelial cells results in acetaldehyde accumulation, which increases the phosphorylation of tyrosine in tight and adherent junctions. It increases the permeability of the intestine to endotoxins, which in turn transfers these to the liver and this results in inflammatory changes in the liver and other organs [42].

Moreover, studies have also found that alcohol and metabolites stimulate the intestinal tract to produce a large number of nuclear transcription factors NF-κB and inducible nitric oxide synthase (iNOS), which increase the intercellular permeability by reacting with tubulin and activating intracellular non-specific protease C. This leads to cytoskeletal phosphorylation, the damage of the microtubule cytoskeleton, and cell conformational changes. It results in the disruption of intestinal barrier functions [42,43]. At the same time, toll-like receptor (TLR) mediates the synthesis and release of cytokines and induces the massive release of inflammatory factors, impairing antiviral infection and systemic immune regulation function. This induces the occurrence of “leak” in ALD patients which facilitates the entrance of endotoxin into the blood circulation [44] (Figure 1). Blood endotoxin can activate Kupffer and inflammatory cells, which inhibit macrophage phagocytosis and stimulate the proliferation of hepatic stellate cells (HSC) by releasing cytokines such as TNF-α, interleukin IL-1, IL-17, CXC chemokines, osteopontin, and inflammatory factors and free radicals [5] (Figure 1). IL-17 can also promote the recruitment of neutrophils by stimulating the production of IL-8 and (chemokine (C-X-C motif) ligand 1) CXCL1 by hepatic stellate cells (HSC) (Figure 1). These activated Kupffer cells and neutrophils release fibrosis-related factors, such as the transforming growth factor beta (TGF-β) and the platelet-derived growth factor [45].

#### 2.3.4. Hepcidin Regulation

The research demonstrates that the iron deposition in liver is associated with ALD and more than 30% of patients with ALD suffer from iron metabolism disorders [46]. Recent studies have shown that even mild alcohol consumption induces the iron stores in the liver [46,47]. Therefore, it is common to observe iron overload in ALD patients. This is mediated by regulatory mechanisms and one of those underlying mechanisms is the alcohol-mediated down-regulation of hepcidin synthesis in liver [46] (Figure 1). Hepcidin plays a key role in iron homeostasis [48,49]. It is considered as the key regulator of iron circulation in mammals [50]. Alcohol down-regulates the expression of hepcidin by directly acting on it rather than regulating the status of the iron in cells [51].

Generally, the overload of iron increases the hepcidin synthesis in the liver as a response to the body’s iron level [48]. However, alcohol exposure down-regulates the expression of hepcidin in the liver despite an iron overload [51]. Therefore, a study has demonstrated that alcohol makes the synthesis of hepcidin insensitive to iron levels in the body [52]. Thus, alcohol has been reported to compromise the molecular mechanisms which protect the body from the damages by iron overload [52].

It is also well-known that iron and alcohol, individually, are capable of inducing oxidative stress and lipid peroxidation [53]. Therefore, both alcohol and alcohol-induced iron enhance the production of free radicals and pro-inflammatory cytokines [54,55]. Moreover, oxidative stress increases the level of transferrin receptor 1 (TfR1) which then promotes the intestinal iron absorption and body iron indices (Figure 1). Altogether, the increased iron absorption and deposition aggravates liver damage [46,56].

### 2.4. Apoptotic Signaling Pathway and Autophagy in ALD

**Figure 2:**
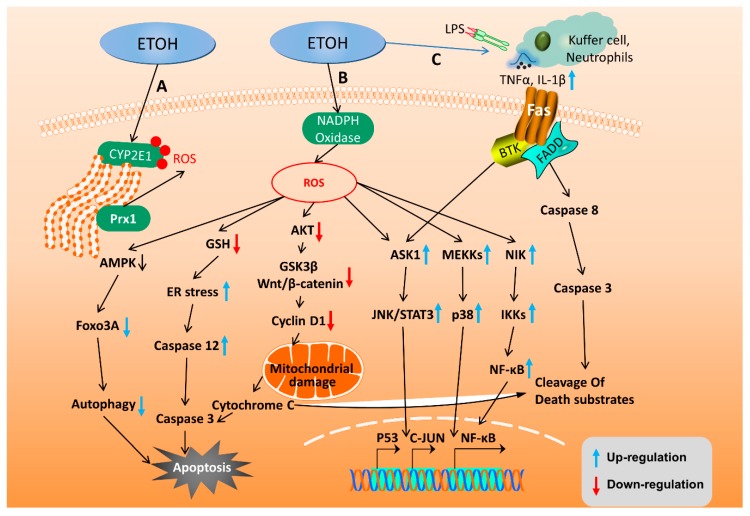
Molecular mechanisms of alcohol-induced apoptosis and autophagy in hepatocytes. (**A**,**B**) Intracellular ROS is produced through the alcohol metabolism via CYP2E1 and NADPH oxidase. These induced ROS level cause hepatocyte apoptosis through AMPK, GSH, AKT, JNK/STAT3, and NF-κB pathways. (**C**) Alcohol consumption activates Kupffer cells to release TNF-α AND IL-1β, which then activates the Fas ligand-dependent caspase pathway to induce hepatocyte apoptosis.

The apoptosis and autophagy of hepatocytes are pathological conditions that accompany ALD. Alcoholic liver toxicity and alcohol metabolism induce oxidative stress and inflammatory reactions which then cause hepatocyte apoptosis and accelerate the progression of liver diseases. Apoptosis induced by mitochondrial damages and ER stress caused by oxidative stress during ALD have been extensively studied. Accumulated ROS due to alcohol metabolism plays an important role in this mitochondria-dependent apoptosis. ROS accumulates in hepatocytes and inhibits the phosphorylation of alpha serine/threonine-protein kinase (AKT) [57], thereby down-regulating cycle G1 protein cyclin D1 through the inhibition of glycogen synthase kinase 3 beta (GSK3β)/Wnt/β-catenin signaling pathway activation in hepatocytes [58]. It causes the cell cycle arrest and activates mitochondria-dependent apoptosis. ROS can also directly activate apoptosis signal-regulating kinase 1 (ASK1) [59], nuclear factor kappa-light-chain-enhancer of activated B (NF-κB), and c-Jun N-terminal kinases (JNK)/P38 to induce mitochondrial-dependent apoptosis [60] (Figure 2).

Moreover, the alcohol metabolism in the gut and liver change the microorganisms in the gut and induce the production of LPS, which in turn increases the inflammatory responses, causing liver inflammation and dysfunctions in lipid metabolism [61]. On the other hand, both ROS and LPS induce the production of TNF-α [62], promote the expression of pro-inflammatory factors IL-1β [7], induce apoptosis by ER stress, induce caspase cascade by activation of JNK/STAT3 and P53 [63], and cause Fas ligand-dependent apoptosis by recruit neutrophils [57] (Figure 2).

Autophagy is the self-destruction of apoptotic cells which degrades the damaged proteins and organelles by lysosomes [64]. Numerous studies have shown that autophagy has inhibitory effects on steatosis, inflammatory response, and hepatocyte apoptosis during ALD [65,66,67,68]. Moreover, research haa shown that various pathways, such as AMPK-Forkhead box O3 (FOXO3A) axis, sorting nexin 10 (SNX10)/chaperone, ALDH2, cannabinoid receptor 2 can induce autophagy to prevent alcoholic liver damage [61,69,70,71,72] (Figure 2).

### 2.5. Genetic Factors of ALD

According to previous reports, more than 95% of chronic alcoholics suffer from alcoholic fatty liver disease. However, only approximately 30% of patients develop severe stages and the potential protective mechanisms behind this are still unclear [73]. Previous studies have concluded that the consumption of different amounts of alcohol in various populations or the different expression levels of ALDH and CYP2E1 enzymes between individuals may result in this variation in disease progression. Recent case-control studies in the United States have shown that the single nucleotide rs738409 polymorphism in the Patatin-like phospholipase domain-containing protein 3 (PNPLA3) gene is closely related to the incidence of alcoholic liver [74]. Additionally, it was reported that single-nucleotide rs738409 polymorphisms are strongly associated with ALD in Han Chinese males [75]. In patients with advanced liver disease, PNPLA3 I148M variant primarily contributs to increase the risk of ALD development into HCC. It also causes a poor prognosis for liver transplantation [76]. Therefore, I148M may act as a genetic indicator for evaluating whether a patient is eligible for liver transplantation.

## 3. Early Diagnosis of ALD

### 3.1. Clinical Symptoms

Early manifestations of ALD in are fatty liver and mild hepatitis. The diagnosis of ALD requires the faithful reflection of patients on their history of chronic drinking to avoid difficulties in diagnosis. The presence of alcoholic liver can generally be judged according to common symptoms, such as fatigue, loss of appetite, bloating, nausea, vomiting, and obesity [77]. Clinically, the most common method to diagnose ALD is the liver function test. It takes the advantage of a 5 to 8 times higher aminotransferase level in blood samples of ALD patients compared to that in a healthy person. Generally, the aspartate transaminase (AST)/alanine transaminase (ALT) ratio can be used to detect ALD, as 70 to 80% of patients have twice the ratio of AST/ALT compared to a healthy person [78].

Gamma-glutamyltranspeptidase (GGT) is another sensitive index to diagnose fatty liver in cases of excessive alcoholism, as it combines the content of triglyceride and cholesterol in serum. However, these indicators cannot specifically diagnose whether liver damage is caused by obesity or alcohol-induced liver damage. Alcoholic liver and non-alcoholic fatty liver also have similar pathology ranges, ranging from simple fatty liver to steatohepatitis and cirrhosis. Therefore, the B-ultrasound test is needed to confirm the presence of fatty liver and the status can be further confirmed by CT test [6,79].

### 3.2. Imaging Examination

B-ultrasound is a commonly used technique in clinical diagnostics. It can prescribe three cases of abdominal ultrasound manifestations. The satisfaction of two of those indicates the diffuse fatty liver. First, is the diffuse enhancement of near field echo of the liver called “bright liver”. Normally, the bright liver echo is stronger than the kidney. Second, the manifestation of gradual echo attenuation in the liver far field. The third is characterized by significantly enhanced near-field echo, an obviously attenuated far field echo, and the tubular structure of the liver. An ultrasound diagnosis of alcoholic liver usually diagnoses the condition of the disease, effectively judges the degree of the disease, and improves the condition in time. Other advantages, such as the simple operation, not causing trauma to the patient, and high safety make the B-ultrasound widely promoted [80].

Computed tomography (CT) is used to diagnose the conditions that reduce the diffuse liver density. Usually, the ratio of CT values of the liver to spleen is reduced with the diffuse liver density in patients. According to studies, the liver/spleen CT ratio is ≤1.0 but >0.7 in mild conditions, ≤0.7 but >0.5 in moderate level conditions, and ≤0.5 in severe conditions [18,77].

The imaging examination is also used to reflect the distribution of liver fat infiltration, roughly determine the degree of diffuse fatty liver, and indicate the presence or absence of cirrhosis. However, it cannot distinguish the simple fatty liver and steatohepatitis separately. Also, it is difficult to detect liver cell steatosis lower than 33% in its grading. It also has to consider that the diffuse liver echo enhancement and density reduction can be seen in other chronic liver diseases [81].

### 3.3. Detection of Specific Biomarkers

Carbohydrate-deficient transferrin (CDT) is a highly specific biomarker for the detection of chronic alcohol abuse. It is the most ideal marker for judging drinking conditions. Generally, the content of alcohol metabolites in the urine 4–5 days after the last alcohol abuse is determined by CDT [82]. Especially during severe AH, it is a fundamental tool to define the severity of the disease and to differentiate AH from decompensated cirrhosis [78]. Another study found that when liver fibrosis or fatty liver is present in patients with alcoholic liver disease, the combination of CDT and other biochemical indicators (such as GGT, AST/ALT ratio) can further improve the accuracy of FibroTest and SteatoTest which is recommended by World Health Organization (WHO) for testing patients with metabolic conditions or who consume excessive amounts of alcohol [83].

Mass spectrometry-based metabolomics analysis is a rapid, non-invasive urine test to identify early stage alcohol-induced liver disease. This permits risk stratification and the treatment of high-risk individuals, before ALD leads to irreversible liver damage and death [84].

## 4. Current Therapies

Mild liver damage caused by alcoholism can be eliminated by long-term abstinence, which is currently the main treatment for ALD. However, treatments for alcoholics depend on the degree of alcohol consumption [8]. On the basis of previous studies, we have highlighted those available therapies and promising approaches to treat ALD patients.

### 4.1. Nutritional Therapy

Alcoholic liver patients often suffer from malnutrition caused by protein damage, which induces bacterial infections. Therefore, it is recommended to supply nutritional support for patients with mild AH by providing high protein, low-fat diets, and balancing the levels of vitamin B, C, K, and folic acid [85]. This nutrient supplement upgrades the conditions of patients as observed in previous studies through short-term diagnosis, such as liver function analysis and histological analysis. Patients with acute alcohol intoxication or high hepatic encephalopathy should protect the respiratory tract, and patients with severe AH usually need a referral rehabilitation program. According to clinical studies, the daily protein intake of ALD patients should be 1.5 g/kg of body weight. Furthermore, it is necessary to supply vitamin B often to patients with ALD and malnutrition, due to the potential risk of Wernicke encephalopathy [5].

### 4.2. Alcohol Withdrawal Therapies

Special alcohol withdrawal drugs are used clinically as adjuvant treatment to treat alcoholics who are addicted to alcohol consumption and who actively drink alcohol. Patients with liver transplantation treatment plans need to obtain liver transplantation qualification through long-term alcohol withdrawal. Disulfiram is an irreversible alcohol dehydrogenase inhibitor that is often used to treat alcoholism. However, it is not advised to use Disulfiram for advanced ALD patients, due to its potentially severe hepatotoxicity [73]. Acamprosate, a medication used along with counseling to treat alcohol dependence, is effective in preventing recurrence, but may have potential liver toxicity [86]. Baclofen, a gamma-aminobutyric acid B receptor agonist, has been found to be effective in maintaining a withdrawal, even in patients with cirrhosis. Additionally, the opioid antagonist naltrexone has been shown to reduce relapse, although its efficacy is moderate [87].

### 4.3. Hormone Related Therapies

Corticosteroids have been used to improve the nutritional status of AH patients. The anti-thyroid drug propylthiouracil also has been evaluated for the treatment of acute AH. However, the analysis of six clinical trials has shown that propylthiouracil does not affect survival time and is associated with adverse reactions [73]. Since ALD is associated with elevated levels of oxidative stress, antioxidants such as vitamin E and silymarin have been investigated and evaluated for the treatment of AH patients in previous studies. Unfortunately, the survival time of patients did not increase [5]. However, another study, which evaluated the potential benefits of the combination of N-acetylcysteine and corticosteroids, showed an increase in patient survival [5].

Losartan is considered a treatment to prevent the development of hepatic fibrosis and progression and regression of fibrosis stage [88,89]. Prednisolone, a steroid medication, is used to inhibit the inflammation of hepatocytes [4]. Although initial trials with oxandrolone showed positive results, it was not confirmed with further studies and no benefit was shown in a meta-analysis [90].

### 4.4. Liver Transplantation

Liver transplantation is the main choice for patients with advanced-stages of ALD. Liver transplantation has a better prognosis for patients with severe alcoholic hepatitis, who are not sensitive to drug treatments [7]. A physiologically functioning liver can be provided to patients with ALD by liver transplantation. Even though it increases the survival and quality of patient’s life, it does not eliminate the underlying alcoholism. Therefore, it may have a potential for relapse [91,92,93].

### 4.5. Targeted Therapies

In this section, we highlight the potential early therapeutic targets and provided promising therapeutic targets in various stages of ALD.

#### 4.5.1. TNF Receptor Superfamily Target Therapies

Several clinical studies have been conducted on the effects of reagents, such as pentoxifylline or S-adenosyl-L-methionine, on AH patients that block TNF-α.

Pentoxifylline, a phosphodiesterase inhibitor that blocks the transcription of TNF-α, has successfully reduced mortality in patients with severe AH, which results in the decreased development of hepatorenal syndrome. Pentoxifylline can be used to treat patients with severe AH who are unable to administer glucocorticoids but it is not effective in the rescue of patients who are not responding to corticosteroids [73].

S-adenosyl-L-methionine (SAMe) is a methyl donor that has been shown to have antioxidant activity and prevents ALD by maintaining the mitochondrial function and further down-regulating TNF-α. Early clinical studies have shown that the use of SAMe can significantly reduce mortality in ALD patients. However, in another randomized controlled trial, there was no evidence to support the efficiency of SAMe in ALD therapy. Therefore, the therapeutic activity of this compound on ALD is yet to be determined. In addition, the clinical efficacy of SAMe as a treatment for ALD should be further established in large-scale research with the help of well-designed trials [73,94].

#### 4.5.2. Antioxidant Signal Targeting Therapies

Activation of antioxidant genes such as Nrf-2, [38] NF-κB, superoxide dismutase, [95,96] catalase, glutathione, peroxidase-1, metallothionein, and peroxiredoxin-1 [97,98] is the targeted treatment for ALD caused by oxidative stress. Furthermore, the drugs inhibit CYP2E1, reduce reactive oxygen species, promote PPARα and activate SIRT1, AMPK, [99] HO-1, [100] and other pathways that can be used for the treatment of ALD.

#### 4.5.3. Targeting the Inhibition of Hepatocyte Apoptosis

The whole process of acute and chronic ALD is accompanied with apoptosis. Clinical trials have shown that various caspase inhibitors can alleviate liver damage and fibrotic or nonalcoholic steatohepatitis in chronic hepatitis C virus (HCV) infected patients. Therefore, its use was suggested for ALD. Moreover, therapeutic, reversible and specific caspase high activity inhibitors are ideal drugs to treat ALD, as side effects can be avoided by inhibiting other cysteines. Caspase cascade can be activated by pathways such as mitochondrial-dependent, FAS/Fas ligand (FASl)-dependent [101] and endoplasmic reticulum pathways.

Also, the activation of upstream anti-apoptotic signaling such as SIRT/FOXO1 [62], PI3K/AKT, Wnt/β-catenin/FOXO3A [58,71] and the inhibition of pro-apoptotic protein P53/P21can inhibit alcohol hepatocyte apoptosis and alleviate liver damage. Furthermore, the regulation of anti-inflammatory signaling IL-10, IL-15 and pro-inflammatory factor IL-6/STAT3 also inhibit hepatocyte apoptosis [102,103]. Studies have also shown that mitogen-activated protein kinase (MAPK) family signaling proteins are highly expressed in ALD [60,104,105], which may serve as a target for inhibiting apoptosis of alcoholic hepatocytes.

#### 4.5.4. MicroRNAs Targeted Therapies

MicroRNAs are a variety of biologically-regulated trimmers that are expressed in various cell types of gut-liver. These MicroRNAs participate in oxidative stress, inflammatory responses, lipid metabolism, and the activation of HSCs by regulating the transcription of target proteins. In the pathogenic process of alcoholic liver, the expression levels of miR-155, miR-132, and miR-182, are significantly up-regulated. However, the microRNAs such as miR-148a, miR-203, and miR122 are downregulated. Therefore, as shown in Table 1, the downregulation or overexpression of specific microRNAs can be used as therapeutic targets for ALD. Moreover, studies have shown that melatonin and intestinal probiotics prevent alcoholic liver by regulating microRNAs [106,107].

### 4.6. Specific Treatment Options for AH

AH belongs to the severe ALD, a pathological stage in which patients have obvious symptoms of liver injury. Unlike alcoholic fatty liver, it requires alcohol abstinence and adjuvant drugs in addition to nutritional support. Furthermore, hospitalization is needed to prevent bacterial infection in severe cases. Many types of drugs have been used currently to manage alcoholic hepatitis patients in the clinic. However, the poor effect of these drugs remains a major challenge to overcome. Researchers are generally focused on studying the pathogenesis and therapeutic targets of AH. In addition, many pharmaceutical companies also are interested in drug development for AH. Therefore, here, we simply sorted out several potential treatment targets of AH.

The pro-inflammatory factor IL-6 is expressed in high levels in patients with AH. Therefore, inhibiting IL-6 is considered a potential target for the treatment of AH. However, a study has shown that IL-6-deficient mice are susceptible to AFL [123]. IL-1 is a multipotent immune regulator in vivo and may drive multiple spontaneous inflammations [124]. IL-1β accelerates the effect on production of neutrophil-associated adhesion factor E-selectin in Gao–Binge animal model [125]. IL-1R antagonists and IL-1β inhibitors can alleviate inflammasomes-dependent ASH [124]. IL-22 is a member of IL-10 cytokine family that controls bacterial infection by activation of STAT3 [126,127]. IL-22 can be used to treat ALD patients because of its antioxidant, anti-apoptotic, and anti-steatogenic, antibacterial, and proliferative effects [126,128]. In addition, IL-22 may be a good therapeutic target that overcomes the promotion of corticosteroid-mediated infection as the IL-22 receptor is expressed only on epithelial cells such as hepatocytes.

The CXC chemokine family includes ELR^+^ or ELR^-^. The members of ELR^+^ CXC family usually attract neutrophils and infiltrate the liver of ALD patients [129]. In AH patients, the expressions of CXCL1, CXCL2, and Chemokine (C-C motif) ligand 20 (CCL20) are increased in the liver [130,131,132] and CXCR1-CXCR5 are considered as the main receptors [133,134]. Moreover, the higher expression levels of IL-8, CXCL5, Gro-γ, and CXCL6 are associated with a worse prognosis of ALD [129]. Therefore, the agents that target CXC chemokines may be developed as therapeutic agents for AH.

The regulation of intestinal flora and attenuation of the LPS-TLR4 signaling pathway are considered as effective treatment strategies of ALD [135,136,137]. Previous studies have reported that intestinal probiotics can promote the expression of alcohol metabolism genes in the intestine. Additionally, it is capable of reducing the expression of TNF-α and TLR4 [138,139], increasing the production of IL-10, and preventing the dysregulation of intestinal microbiota through the addition of probiotics, [140] such as *Lactobacillus rhamnosus GG, Akkermansia muciniphila*, [141] *Lactobacillus fermentum* [142]. The addition of probiotics also can improve the intestinal permeability by regulating miRNA production and promoting intestinal occludin protein expression [107]. Rifaximin, a non-absorbable antibiotic that alters the intestinal microbiota, is known to be effective in the treatment of hepatic encephalopathy in clinical [5,78]. Inhibition of TLR4 signaling attenuates alcohol-induced liver injury and provides a therapeutic target for the treatment of alcoholic liver. According to studies, it is useful for the treatment of ALD by modifying TLR4 inhibitor or inhibiting LPS-induced TNF-α production.

Osteopontin is an extracellular matrix protein which is significantly up-regulated in ALD patients [143]. It promotes the pathogenesis of ALD by activating HSC. Therefore, the expression level of osteopontin positively relates with the severity of disease [144]. Early studies have shown that blocking osteopontin is effective in improving ALD [145]. However, it also reduces the production of TNF-α by binding LPS in the early stage of ALD [145]. The loss of osteopontin promotes the infiltration of neutrophils in the liver [146] and also leads to the transfer of hematopoietic stem cells into the liver and induces ALD by increasing iron content in liver [147]. Therefore, the mechanism of differential expression of osteopontin needs further study.

Endogenous cannabinoids mediate lipid metabolism and inflammatory responses in the liver and participate in ALD through the cannabinoid receptor (CB) 1 and cannabinoid receptor (CB) 2 regulatory signaling pathways [148]. Research has shown that CB1-deficient mice are resistant to alcoholic liver [149], and the pro-inflammatory response caused by CB1 can be negatively regulated by CB2 [150]. Moreover, it has shown that CB1 antagonists and CB2 agonists can protect alcohol-induced liver injury through different pathways [151,152]. However, the usage of CB1 antagonists has been limited in the treatment of liver diseases due to its neuropsychiatric side effects. Therefore, peripherally restricted CB1 antagonists have been actively explored recently. The inhibition of the liver CB1 receptor transcriptional regulator and estrogen-related receptor, has been reported to control alcohol-induced liver damages [153]. It may provide therapeutic benefits for ALD patients synergistically with CB2 agonists [69,152].

### 4.7. Pluripotent Mesenchymal Stem Cells for the Treatment of Liver Fibrosis

**Figure 3:**
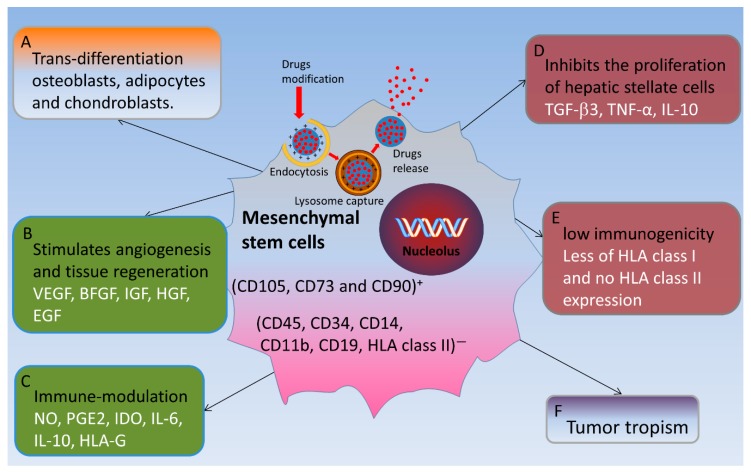
Possible interventions of mesenchymal stem cells in ALD. Interventions of MSCs consist of (**A**) trans-differentiation into parenchymal cells (**B**) induction of endogenous regeneration (**C**) modulation of inflammation and (**D**) decreased liver fibrosis via inhibiting the proliferation of hepatic stellate cells. (**E**,**F**) Specific characteristics of MSCs which facilitate the intervention of MSCs.

Liver fibrosis is the final stage of many chronic liver diseases. Once patients are diagnosed with alcoholic liver fibrosis/cirrhosis, alcohol withdrawal only can slow down the onset of the disease. According to previous studies, liver transplantation therapy remains the only treatment for alcoholic liver fibrosis. However, with the development of stem cell therapy research, the therapeutic potential of MSC, HSC, and BMC in liver regenerative medicine was compared. MSCs have low immunogenicity and tumor tropism in addition to properties such as cell proliferation, self-renewal, and self-differentiation. MSCs can inhibit the inflammatory response, reduce hepatocyte apoptosis, increase hepatocyte regeneration, reverse liver fibrosis, and enhance liver metabolism. Due to these characteristics, MSCs can alleviate the condition of liver fibrosis and cirrhosis. MSCs also carry and transform the chemotherapeutic drugs into the liver cancer site to release anti-cancer components and promote liver regeneration by escaping from immune surveillance system in the body [154]. Although MSC population is rare (<0.01% of monocytes present in the bone marrow), they can easily perform in vitro amplification. Therefore, MSCs are considered ideal candidates for cell therapy [9] (Figure 3).

Few identification criteria have been reported for MSCs. First, it must be plastic-adhered when stored under standard culture conditions. Second, it must express CD105, CD73, and CD90, and be free from the expression of CD45, CD34, CD14, CD11b, CD19, and human leukocyte antigens surface molecules II. Third, it must have the ability of differentiation into osteoblasts, adipocytes, and chondroblasts under in vitro differentiation culture conditions [155,156] (Figure 3).

#### 4.7.1. Functional with Low Immunogenicity and Inhibition of Inflammatory Responses

MSCs can be obtained from donors without serious complications, since MSCs express a small amount of Human leukocyte antigen (HLA) class I and do not express HLA class II molecules, which allows them to evade allogeneic immune response [157]. In addition, MSC transplantation has been tested, with promising results, in several clinical trials, and is a possible alternative approach for thetreatment of patients with liver diseases. MSCs can reduce collagen deposition in patients with alcoholic liver cirrhosis [158,159]. MSCs can directly inhibit the activity of CD8^+^ cytotoxic T lymphocytes by inhibiting their proliferation after antigen stimulation, and indirectly inhibit the activity of CD8^+^ cytotoxic T lymphocytes by increasing the relative proportion of CD4^+^T helper-2 (TH2) lymphocytes and CD4^+^ regulatory T lymphocytes. Since B lymphocyte activation is largely dependent on T cells, the effect of MSCs on T lymphocytes indirectly relates to the inhibition of B cell functions. In addition, MSCs also have a direct effect on B lymphocytes through cell-cell contact and the secretion of paracrine molecules [160] (Figure 2).

#### 4.7.2. Tumor Tropism

The therapeutic potential of MSCs is also based on their inherent ability of being home to sites of inflammation after tissue injury. Damaged tissues release several molecules that interact with different receptors expressed by MSCs. It gives MSCs the ability to migrate through the endothelial cell layer and be attached and retained in ischemic tissue rather than in distal or intact tissues. Although the mechanisms driving this property are not fully understood, damaged tissues may express specific receptors or ligands that promote the transport, adhesion, and infiltration of MSCs to damaged sites, similar to white blood cells [161,162] (Figure 3).

#### 4.7.3. Promotes the Proliferation and Regeneration of Hepatocytes

MSCs can differentiate into hepatocyte-like cells in vitro and in vivo and secrete a wide range of cytokines, including IL-10 which inhibits the apoptosis of hepatic parenchymal cells, vascular endothelial growth factor (VEGF), and basic fibroblast growth factor (BFGF), insulin-like growth factor (IGF), hepatocyte growth factor (HGF), epidermal growth factor (EGF), chemokines, transforming growth factor β3 (TGF-β3), and TNF-α [163,164]. These secretions inhibit the proliferation of hepatic stellate cells, reduce the inflammation and fibrosis of damaged tissues, improve impaired tissue, prevent parenchymal cell apoptosis, provide nutritive support for damaged tissues, and induce local precursor cell proliferation and differentiation by changing the microenvironment to stimulate angiogenesis and tissue regeneration [156]. In addition, MSCs can also express various soluble factors such as nitric oxide (NO), prostaglandin E2 (PGE2), indoleamine 2, 3-dioxygenase (IDO), IL-6, IL-10, and human leukocyte antigens G (HLA-G). These soluble factors regulate the proliferation and function of a variety of immune cells, and they have also been shown to induce regulatory T cells (Treg) [162,165] (Figure 3).

### 4.8. Research Studies on the Effects of Natural Plant Extractions for ALD

Treatment with natural plant extractions for ALD has gained attention, since an effective treatment for ALD is still limited. The low toxicity and easy absorption nature of herbal medicines and food extracts is advantageous in these treatments. In previous in vitro and in vivo studies, a variety of natural medicines and plant extracts have shown anti-oxidation and anti-inflammatory effects and the ability to regulate fat metabolism.

Researchers have found that food extracts such as garlic, betaine, anthocyanin, and resveratrol have protective effects on the alcoholic liver as shown in Table 2. Furthermore, they have found that herbal plant extracts have the effect of protecting the liver and clearing fat, as shown in Table 3. A large amount of active compounds were identified to prevent alcoholic liver from herbal plants such as Panax genus, salvia, Pueraria, astragalus, ginger, Silybum marianum, and licorice. Moreover, traditional Chinese medicine formula Zhi-Zi-Da-Huang, Qinggan Huoxue Recipe and Hugan Qingzhi tablet (HQT) have inhibitory effects on ALD and may be involved in multiple stages of ALD [166,167,168].

## 5. Conclusions and Future Directions

ALD is the leading cause of global advanced liver disease. Significant progress has been made in understanding the pathogenesis of animal models at the experimental level. Additionally, few therapeutic targets have been identified using transformation studies of human liver samples. However, the translation of basic research and translational research results into new therapies has been modest. Future efforts should be focused on identifying the main factors that contribute to the disease in patients with moderate and severe ALD to develop new therapies. For the terminal phase of ALD, the only effective treatment is liver transplantation [207]. However, it also still has several problems, including the lack of a donor source, immune rejection, and high surgical costs. However, the use of cell therapy is gaining attention, and mesenchymal stem cells (MSCs) appear to be promising cell types for the treatment of liver fibrosis. MSCs have a variety of differentiation capabilities allowing them to migrate directly into damaged tissues and differentiate into hepatocyte-like cells. In addition, MSCs release various growth factors and cytokines to increase hepatocyte regeneration, resolve liver fibrosis, and regulate inflammation and immune response [5].

## Figures and Tables

**Table 1:** Involvement of MicroRNAs in ALD.

MicroRNAs	Target	Dysregulation	References
miR-203	Lipin1	Decreased	[108]
miR-122	Lipid metabolism: HMGCoA, ApoE, MTTP, PGC1a, HIF1A; Fibrosis: P4HA1; Hepatocellular cancer: Igf1R, ADAM10, cyclin G1, KLF6	Decreased/Increased	[109,110,111]
miR-30e	UCP, ATP	Decreased	[112]
miR-19b	miR-17-92	Decreased	[113]
miR-497	Btg2, Yy1	Decreased	[114]
miR-148a	FOXO1, TXNIP	Decreased	[115]
miR-217	SIRT1/Lipin1	Increased	[116]
miR-214	GSR, CYP450 OR	Increased	[117]
miR-132	Neuroimmune microRNAs	Increased	[111,118]
miR-155	FABP4, LXRα, ACC1, LDLR	Increased	[109,111,118,119]
miR-29a; Let-7f; miR-340	ASH	Increased	[120]
miR-17-92	Fibrosis	Increased	[113]
miR-200a	ZER2	Increased	[121]
miR-34a	SIRT1	Increased	[122]

**Table 2:** Active substances extracted from food to prevent alcoholic liver.

Names	The Sources	Target	References
Resveratrol	Grapes, red wine, peanuts and berries.	HIF-1α, Oxidative stress	[169,170,171]
Diallyl trisulfide	Garlic	Nrf-2/HO-1, Steatosis	[172,173,174,175]
Anthocyanin	Purple potato, wild grape	Anti-inflammatory, Oxidative stress, Steatosis	[62,176,177,178,179]
Tomato powder	Tomato	CYP2E1	[180]
Fisetin	Strawberry, apple, persimmon, lotus root, and onion	NOX4, Adiponectin, AMPK	[181]
Ginger-derived nanoparticles	Ginger	Nrf-2	[182]
β-caryophyllene	Plant-derived food additive	Cannabinoid 2 receptors	[183]
lychee pulp phenolic	Lychee	Fatty acid β-oxidation, Hepatocyte apoptosis	[184]

**Table 3:** Active substances extracted from herbs for preventing alcoholic liver.

Names	The Sources	Target	References
Ginsenosides	*Ginseng*	Steatosis, oxidative stress	[185,186,187]
Salvianolic acid	*Salvia miltiorrhiza*	SIRT1, P38, NF-κB	[188,189,190]
Puerarin	*Pueraria lobata*	PPARα, AMPK	[191,192,193]
Baicalin	*Scutellaria baicalensis*	Hedgehog Pathway, Oxidative stress, Inflammation	[194,195,196]
Curcumin	*Curcuma longa*	NF-κB, Nrf-2, Cytochrome c, Lipid peroxidation	[197,198,199]
Glycyrrhizic acid	*Glycyrrhiza uralensis*	Glutathione, TNF-α	[200,201,202]
Berberine	*Coptis chinensis*	PPARα, HNF4α	[203]
Ligustrazine	*Ligusticum chuanxiong*	Nrf-2, Hepatic steatosis	[204]
Honokiol and magnolol	*Magnolia officinalis*	AMPK/SREBP-1c, CB2	[205,206]

## Abbreviations

ACC	Acetyl-CoA Carboxylase
ADH	Alcohol Dehydrogenase
AFL	Alcoholic Fatty Liver
AH	Alcoholic Hepatitis
AKT	Serine/threonine-protein kinase B
ALD	Alcoholic Liver Disease
ALDH	Acetaldehyde Dehydrogenase
ALT	Alanine Transaminase
AMPK	AMP-activated protein kinase
ApoE	Apolipoprotein E
ASK	Apoptosis Signal-regulating Kinase 1
AST	Aspartate Transaminase
ATP	Adenosine Triphosphate
BFGF	Basic Fibroblast Growth Factor
BMC	Bone Marrow Cells
Btg2	B-cell translocation gene 2
CAT	Catalase Endoplasmic Reticulum
CB	Cannabinoid Receptor
CCL20	Chemokine (C-C motif) Ligand 20
CDT	Carbohydrate-Deficient Transferrin
CXCL	Chemokine (C-X-C motif) Ligand 1
CYP2E1	Cytochrome P450 2E1
CYP450 OR	Cytochrome P450 oxidoreductase
DAMPs	Damage-Associated Molecular Patterns
EGF	Epidermal Growth Factor
Egr-1	Early growth response protein 1
ER	Endoplasmic Reticulum
FABP4	Fatty Acid Binding Protein
FOXO3A	Forkhead box O3
GGT	Gamma-Glutamyltranspeptidase
GSK3β	Glycogen Synthase Kinase 3 beta
GSR	Glutathione reductase
HCV	Hepatitis C Virus
HGF	Hepatocyte Growth Factor
HIF1α	Hypoxia-Inducible Factor 1-alpha
HLA	Human Leukocyte Antigen
HMGCoA	3-hydroxy-3-methyl-glutaryl-coenzyme A reductase
HO-1	Heme Oxygenase
HSC	Hepatic Stellate Cell
IDO	Indoleamine 2,3-dioxygenase
IGF	Insulin-like Growth Factor
Igf1R	Insulin-like growth factor 1 receptor
IL	Interleukin
JNK	C-Jun N-terminal kinases
iNOS	Inducible nitric oxide synthase
KLF6	Kruppel-Like Factor 6
LDLR	Low-Density Lipoprotein Receptor
LPS	Lipopolysaccharides
LXRα	Liver X Receptor alpha
MAPK	Mitogen-Activated Protein Kinase
MSC	Mesenchymal Stem Cell
MTTP	Microsomal Triglyceride Transfer Protein
NAD	Nicotinamide Adenine Dinucleotide
NF-κB	Nuclear Factor kappa-light-chain-enhancer of activated B
Nrf-2	Nuclear factor erythroid 2-related factor 2
PGC1a	Peroxisome proliferator-activated receptor gamma coactivator 1-alpha
PGE2	Prostaglandin E2
PNPLA3	Patatin like phospholipase domain containing protein 3
PPARα	Peroxisome Proliferator-Activated Receptor alpha
P4HA1	Prolyl 4-hydroxylase subunit alpha 1
ROS	Reactive Oxygen Species
RNS	Reactive Nitrogen Species
SAMe	S-adenosyl-L-methionine
SIRT1	Sirtuin 1
SNX10	Sorting Nexin 10
STAT3	Signal Transducer and Activator of Transcription 3
TGF-β3	Transforming Growth Factor beta 3
TfR1	Transferrin Receptor 1
TLR4	Toll-like Receptor 4
TNF-α	Tumor Necrosis Factor alpha
Treg	Regulatory T cells
Trx	Thioredoxin
TXNIP	Thioredoxin Interacting Protein
UCP	Uncoupling Protein
VEGF	Vascular Endothelial Growth Factor
Yy1	Yin Yang 1
ZER2	Zinc finger E-box binding homeobox 2
